# Time-resolved study on signaling pathway of photoactivated adenylate cyclase and its nonlinear optical response

**DOI:** 10.1016/j.jbc.2023.105285

**Published:** 2023-09-22

**Authors:** Yusuke Nakasone, Hiroto Murakami, Shunrou Tokonami, Takashi Oda, Masahide Terazima

**Affiliations:** 1 Department of Chemistry, Graduate School of Science, Kyoto University, Kyoto, Japan; 2 Department of Life Science and Research Center for Life Science, College of Science, Rikkyo University, Tokyo, Japan

**Keywords:** photobiology, photoreceptor, protein chemistry, enzyme mechanism, kinetics, diffusion, nonlinear optical response

## Abstract

Photoactivated adenylate cyclases (PACs) are multidomain BLUF proteins that regulate the cellular levels of cAMP in a light-dependent manner. The signaling route and dynamics of PAC from *Oscillatoria acuminata* (OaPAC), which consists of a light sensor BLUF domain, an adenylate cyclase domain, and a connector helix (α3-helix), were studied by detecting conformational changes in the protein moiety. Although circular dichroism and small-angle X-ray scattering measurements did not show significant changes upon light illumination, the transient grating method successfully detected light-induced changes in the diffusion coefficient (diffusion-sensitive conformational change (DSCC)) of full-length OaPAC and the BLUF domain with the α3-helix. DSCC of full-length OaPAC was observed only when both protomers in a dimer were photoconverted. This light intensity dependence suggests that OaPAC is a cyclase with a nonlinear light intensity response. The enzymatic activity indeed nonlinearly depends on light intensity, that is, OaPAC is activated under strong light conditions. It was also found that both DSCC and enzymatic activity were suppressed by a mutation in the W90 residue, indicating the importance of the highly conserved Trp in many BLUF domains for the function. Based on these findings, a reaction scheme was proposed together with the reaction dynamics.

Adenylate cyclases (ACs) are an important class of enzymes that catalyze the synthesis of cAMP, which is a critical second messenger involved in various intracellular signaling processes in both prokaryotes and eukaryotes. Because of the importance of this function, the reaction schemes of ACs have attracted considerable interest. For revealing the reaction scheme, ACs, in which reaction can be initiated by light, are useful and appropriate. Therefore, photoactivated ACs (PACs), which produce cAMP upon light illumination, are attractive targets (1, 2, 3, 4, 5, 6, 7, 8, 9). PAC was initially found in the unicellular flagellate *Euglena gracilis* (euPAC), in which the activity of the AC domain is regulated by blue light using flavin adenine dinucleotide (BLUF) domains (1, 2). Owing to the involvement of cAMP in diverse intracellular signaling pathways across different organisms, euPACs have been utilized to control biological functions in several organisms (10, 11, 12).

Subsequently, smaller PACs were found in the photosynthetic cyanobacterium *Oscillatoria acuminata* (OaPAC) and the sulfide-oxidizing bacterium *Beggiat*oa sp. (bPAC) (3, 5, 13). These smaller PACs consist of a BLUF domain and an AC domain. The smaller size of these PACs is beneficial for facilitating their delivery and expression in various systems; thus, they have been used to control various biological responses such as sperm motility in mice, swimming behavior in zebrafish, and axonal growth in rat hippocampal neurons, etc. (3, 5, 14, 15, 16, 17, 18, 19, 20, 21) In addition to the smaller size, since the activity of OaPAC in the dark state is the lowest among the PAC proteins and is substantially increased (20∼100 fold) upon exposure to light (4, 22), OaPAC can be used for the precise control of light-induced cAMP production. Furthermore, some mutated variants of OaPAC have been used to enhance the enzymatic activity to improve its performance in optogenetics (23, 24). Therefore, understanding the reaction mechanism of OaPAC becomes more important.

Although euPAC is unstable in a solution, full-length OaPAC (FL-PAC) and bPAC are stable for production and purification, which is important for studying the fundamental principles of communication between the sensor and effector domains. The crystal structures of OaPAC and bPAC have been reported in the dark and light states, revealing a high degree of similarity between the two proteins (3, 4, 6). Figure 1*A* illustrates the crystal structure of OaPAC in the dark state (3). OaPAC forms a homodimer, with the BLUF and AC domains connected by a relatively long helix known as the α3-helix. This α3-helix serves as a site for dimerization and is believed to be involved in signal transduction, as mutations in the helix suppress the light-dependent increase in enzymatic activity (3). The crystallographic structure has revealed local conformational changes near the chromophore, facilitated by hydrogen bonding networks and chromophore migration (4). However, the conformational changes in the protein moiety distant from the chromophore are very minor, at least in the crystal. The only observed relative motions between the superposed BLUF and AC domains are a rotation of 1.3° and a screw translation of 0.13 Å, and the active site of the AC domain has shown no conformational changes (4), although crystal packing effects might restrict other possible motions. The reaction scheme should be studied in solution.

**Figure 1:**
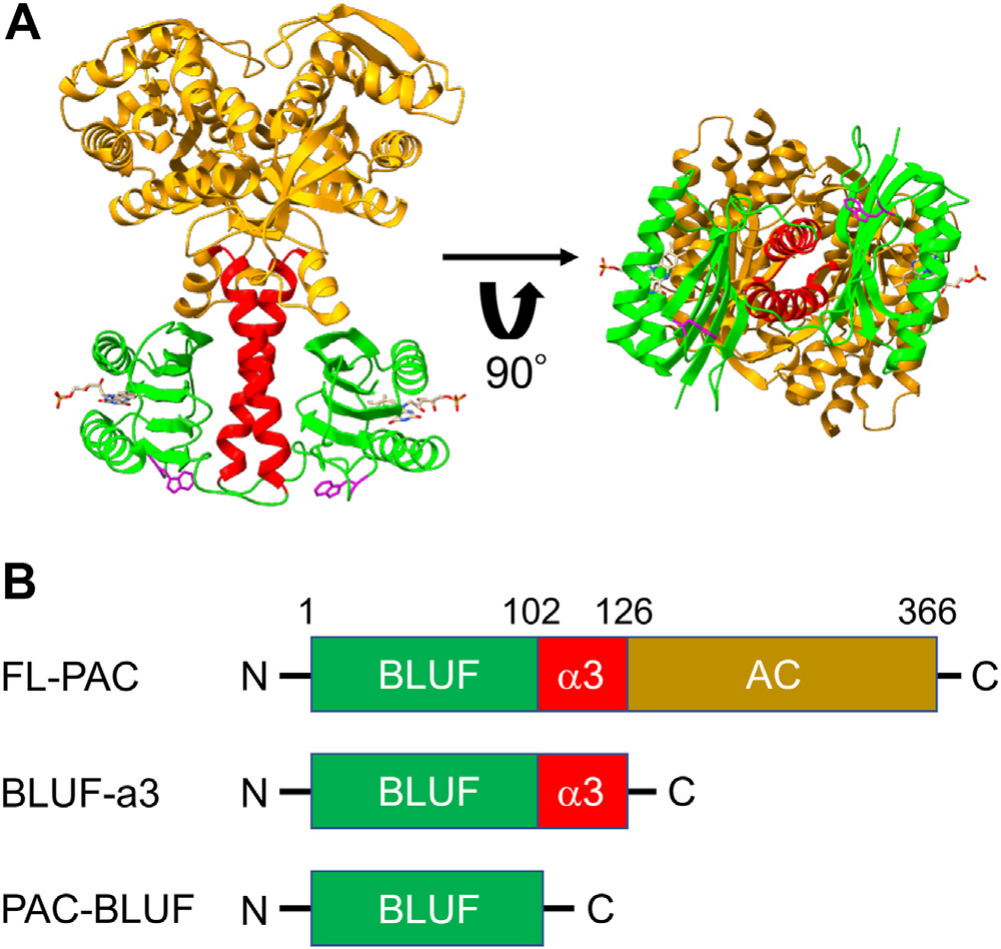
**Crystal structure of OaPAC and constructs used in this study.***A*, crystal structure of OaPAC in the *dark* state (PDB ID: 4yut). The PAC-BLUF domains, α3-helices, and AC domains are shown in *green*, *red*, and *brown*, respectively. The chromophore FMN and Trp90 are shown as *sticks*. *B*, the domain structures of the constructs used in this study are illustrated. AC, adenylate cyclase; OaPAC, PAC from Oscillatoria acuminata; PAC, photoactivated adenylate cyclase.

Extensive studies have been conducted on the photochemistry of the BLUF domain so far (22, 25, 26, 27, 28, 29, 30, 31, 32, 33, 34, 35, 36, 37, 38, 39, 40, 41, 42, 43, 44, 45, 46). Upon photoexcitation of the chromophore, a proton-coupled electron transfer occurs with a nearby tyrosine residue, leading to a change in the hydrogen bonding network involving the tyrosine and glutamine residues (38). This change results in a red-shift in the absorption spectrum, and its kinetics have been investigated by the transient absorption (TrA) technique for several BLUF proteins (31, 32, 33, 35, 45). Although most studies have shown very fast photochemistry of the chromophore within 1 ns, slower dynamics on a millisecond timescale have been discovered recently in many BLUF proteins (43). According to that study, the red-shift of the absorption spectrum of OaPAC occurs *via* two processes with time constants of 2.3 and 36 ms, besides the ultrafast reaction within 1 ns (43). The amplitudes of the slow components are dependent on the presence or absence of the AC domain or the α3-helix (43), suggesting that these reaction phases may reflect the signaling process to the functional domain. The slow processes disappear upon substitution of a highly conserved Trp residue (W90 in OaPAC) with Ala (43), suggesting a critical role of Trp in transmitting the light signal for this function. Despite the accumulated information on the photochemistry of the chromophore, the activation mechanisms of the functional domains remain unclear owing to limited information on structural changes far from the chromophore.

This study aimed to understand the signaling pathways and reaction dynamics of OaPAC by detecting conformational changes in the protein moiety. Various techniques were employed, including transient grating (TG), CD, and small-angle X-ray scattering (SAXS). These techniques enabled the monitoring of protein reactions in the solution phase. In particular, the TG method allowed the observation of both intramolecular and intermolecular reactions by monitoring changes in the diffusion coefficient (*D*) in the time domain (47, 48, 49, 50, 51, 52, 53), known as a diffusion-sensitive conformational change (DSCC). Using these methods, the photoreactions of FL-PAC and two shorter constructs (Fig. 1*B*), one containing only the BLUF domain (PAC-BLUF: 1–102 amino acid residues) and the other consisting of the PAC-BLUF domain with the α3-helix (BLUF-α3: 1–126 amino acid residues), were investigated. Furthermore, unique light intensity dependence on the reaction and catalytic activity was discovered, and using this dependence, the correlation between the observed DSCC and the enzymatic reaction was investigated, and it was found that the reaction observed by the *D*-change is relevant to the activation of OaPAC. In addition, importance of the conserved Trp residue (Trp90) in enzymatic reactions was examined by replacement of this residue with Ala (W90A). Based on these results, the reaction scheme was discussed and it is proposed that OaPAC functions as a nonlinear light intensity sensor that is exclusively activated under strong light conditions.

## Results

### Absorption changes

Fig. S1*A* shows the absorption spectra of FL-PAC, BLUF-α3, and PAC-BLUF. These samples exhibited the characteristic absorption spectra of the BLUF proteins, with a redshift of approximately 10 nm upon light illumination. The thermal recovery process was monitored by measuring the changes in absorption at 495 nm (Fig. S1*B*). These decay curves were fitted using a single exponential function for BLUF-α3 and PAC-BLUF and a double-exponential function for FL-PAC. The time constants are listed in Table 1. It was observed that the recovery rates of FL-PAC and BLUF-α3 were slower, suggesting that the α3-helix and AC domain stabilize the light-adapted state of the BLUF domain.

**Table 1:** Reaction rates and diffusion coefficients of PAC-BLUF, BLUF-α3, FL-PAC, W90A

Sample	*Rate constants*			*Diffusion coefficient* (10^-11^ m^2^/s)			
	(*k*_1_)^−1^ (ms)	(*k*_2_)^−1^ (ms)	*Thermal recovery* (s)	Reactant	I_1_	I_2_	Product
PAC-BLUF	2.8	ND	2.7 ± 0.2	9.2 ± 0.1	9.1 ± 0.3	ND	9.1 ± 0.1
BLUF-α3	4.0	31	2.9 ± 0.1	8.3 ± 0.1	8.3 ± 0.1	7.5 ± 0.2	8.0 ± 0.1
FL-PAC	2.3	36	1.8 ± 0.1, 5.1 ± 0.2	6.2 ± 0.1	6.2 ± 0.1	6.1 ± 0.1	5.3 ± 0.2
W90A	ND	ND	8.2 ± 0.2	6.2 ± 0.2	ND	ND	6.1 ± 0.1

ND, not determined.

### TG measurements

**Figure 2:**
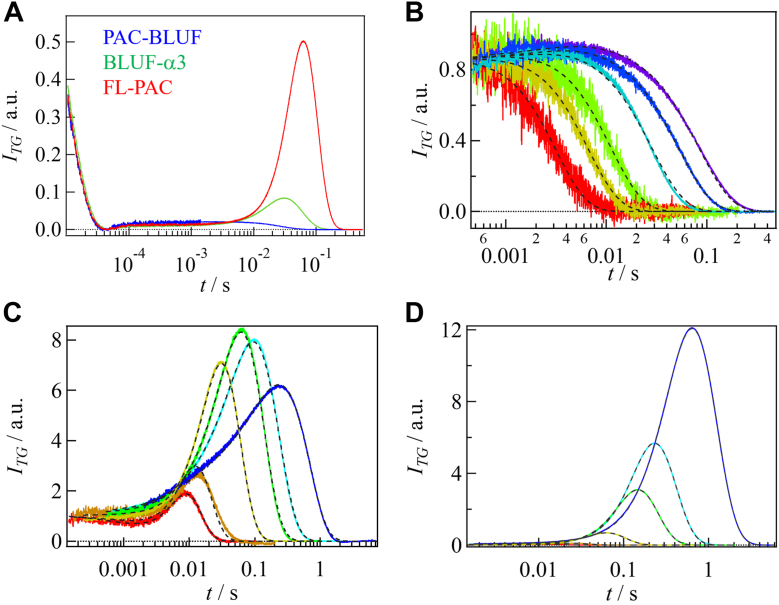
**TG signals of FL-PAC, BLUF-α3, PAC-BLUF, and grating wavenumber dependences of their diffusion signals.***A*, TG signals of FL-PAC (*red*), BLUF-α3 (*green*), and PAC-BLUF (*blue*) at *q*^2^ = 7.2 × 10^11^ m^–2^ and a concentration of 80 μM. The molecular diffusion signals obtained at various *q*^2^ are shown for (*B*) PAC-BLUF, (*C*) BLUF-α3, and (*D*) FL-PAC. The *q*^2^-values are 0.80, 1.4, 4.0, 11, 21, and 44 × 10^11^ m^–2^ from *left* to *right* for all samples. The best-fitted curves based on Scheme 1 (Equation 2) for PAC-BLUF and Scheme 2 (Equation 3) for BLUF-α3 and FL-PAC are shown with *broken black lines*. The intensities of the diffusion peaks of the TG signals roughly represent diffusion changes, which reflect conformation changes. The *q*^2^-dependence indicates the time dependence of the changes. PAC, photoactivated adenylate cyclase; TG, transient grating.

Figure 2*A* shows the TG signals of FL-PAC, BLUF-α3, and PAC-BLUF at a square of grating wavenumber *q*^*2*^ = 7.2 × 10^11^ m^–2^ and a concentration of 80 μM. The signal increased rapidly, followed by a decay within 50 μs after photoexcitation. This decay signal was attributed to the thermal grating signal because its decay rate agreed with *D*_*th*_*q*^*2*^, where *D*_th_ is the thermal diffusivity of the solution. Therefore, the TG signals are expressed by Equation 1 in the Experimental procedures section. The second term of Equation 1, *δn*_spe_(*t*), represents the TG signal in the slower time range of 1 to 200 ms. These slower components were mainly attributed to protein diffusion (diffusion signal) as the time scales of the signals varied depending on *q*^*2*^ (Fig. 2, *B*–*D*). Notably, significant differences in the shape and intensity of the diffusion signal were observed depending on the samples, that is, the signal of PAC-BLUF monotonically decayed, whereas that of BLUF-α3 and FL-PAC exhibited rise-and-decay.

The shape and intensity of the diffusion signal should be strongly influenced by changes in *D* associated with the photoreaction. When *D* does not change by the reaction, a monotonically decaying signal should be observed. However, when *D* changes, a rise-decay profile is expected (49, 50, 53). For PAC-BLUF, the monotonically decaying signal indicates no change in *D*, whereas the observed rise and decay signals for BLUF-α3 and FL-PAC are clear indications of significant changes in *D*. Because the refractive index change of the thermal grating signal (*δn*_th_) is negative under the present experimental conditions, it was found that the signs of the rise and decay components were negative and positive, respectively. Therefore, the rise and decay components were assigned to the diffusion of the reactant and product, respectively, that is, the photoproduct diffused more slowly than the reactant.

In Figure 2*B*, the diffusion signals of PAC-BLUF at different *q*^*2*^ values are shown. No significant changes in the shape and intensity of the signal in any time range indicated no noticeable changes in *D* within the observation time window (1 ms to 1 s). A weak rising signal was observed before the diffusion signal, and its time constant was independent of *q*^*2*^. Therefore, this weak rising component was attributed to the reaction phase rather than a diffusion signal. To analyze the signals, the following reaction scheme was employed, which included an intermediate species (I) before the final product:where *k*_1_ represents the rate constant of the reaction phase. In this case, the time dependence of *δn*_spe_(*t*) is given by Equation 2. The obtained signals were successfully reproduced based on this scheme. The determined *D* values and reaction rate constants are listed in Table 1.

The observed minor change in *D* for PAC-BLUF suggested no significant conformational change in the BLUF domain. Previously, a red-shift in the absorption spectrum with a rate constant of (2.8 ms)^–1^ was reported for PAC-BLUF, and this rate is identical to *k*_1_ in this study (43). Therefore, the rising signal may reflect the absorption change. The *D*-values are reasonable compared to those expected for the dimer, rather than the monomer (54, 55), indicating that PAC-BLUF exists as a dimer in both dark and light states (monomer molecular mass of PAC-BLUF: 12 kDa). This dimeric form was confirmed using size-exclusion chromatography (SEC) analysis (SI-2, Fig. S2).

In Figure 2*C*, the *q*^2^ dependence of the diffusion signal of BLUF-α3 is shown. The intensity of the diffusion signal initially increased and then decreased with decreasing *q*^2^. This behavior indicates the presence of at least two reaction phases in the observation time window, that is, *D* initially decreased significantly and then increased. Therefore, the following reaction scheme, which involves two intermediate species (I_1_ and I_2_) before the final product, was used to analyze the data:

In this case, the time dependence of *δn*_spe_(*t*) is given by Equation 3. Because previous TrA measurements revealed two reaction phases on a millisecond timescale with rate constants of (4.0 ms)^–1^ and (31 ms)^–1^ (43), these rates were fixed for *k*_1_ and *k*_2_ in the analysis to reduce parameter ambiguities. The signals were successfully reproduced using Equation 3, and the obtained parameters are summarized in Table 1. A significant decrease in *D* was followed by an increase. The obtained *Ds* are consistent with the dimeric form of BLUF-α3 (monomer molecular mass: 15 kDa) (54, 55), and this assignment was confirmed using SEC (SI-2). Since the *D*-change of PAC-BLUF is almost negligible, the observed *D*-change for BLUF-α3 is attributed to the movement of the α3-helix.

The diffusion signal of FL-PAC was found to depend on the excitation light intensity as described later. Although the excitation light intensity was controlled to be almost constant (∼100 μJ/pulse) for measurements at various *q*^2^ values, the diffusion signal intensity of FL-PAC exhibited a monotonous increase with decreasing *q*^2^ (Fig. 2*D*). This *q*-dependence should be analyzed based on a proposed reaction scheme. Since two reaction phases were previously reported within the observation time window using the TrA method (43), we used the Scheme 2 for the analysis. The rate constants *k*_1_ and *k*_2_ were fixed by the values of (2.3 ms)^–1^ and (36 ms)^–1^. The signals were reproduced well with these restrictions (Figs. 2*D* and S3*A*) (43), and *D*s were determined (Table 1). It is notable that *D*_I2_ is slightly smaller than *D*_I1_. For confirming the small change from *D*_I1_ to *D*_I2_, we also tried to fit the *q*-dependent signals by assuming *D*_I1_ = *D*_I2_. However, the residues of the fitted curves exhibited systematic and relatively large deviations by this assumption (Fig. S3*B*). Hence, we conclude that *D*_I1_ is slightly different from *D*_I2_ and the Scheme 2 is appropriate for the reaction scheme. In general, the profile of the diffusion signal is sensitive to the change in *D*, and a small change in *D* can be determined by the analysis of one data-set of the diffusion signals (54, 56, 57), whereas the error range shown in Table 1 was determined from the variations of absolute values of *D*s for different batches of samples. Hence, the small change from *D*_I1_ to *D*_I2_ is not in contradiction with this error range.

(Scheme 2) $$R\overset{h\nu }{\to }I_{1}\overset{k_{1}}{\to }I_{2}\overset{k_{2}}{\to }P$$

The results revealed a slight decrease in *D* during the initial step, followed by a more pronounced decrease. This behavior differed from that for BLUF-α3, indicating that a significant conformational change occurs in the AC domain upon photoexcitation. The obtained *D*s are consistent with the dimeric form of FL-PAC (monomer molecular mass: 41 kDa), as confirmed by SEC (SI-2).

**Figure 3:**
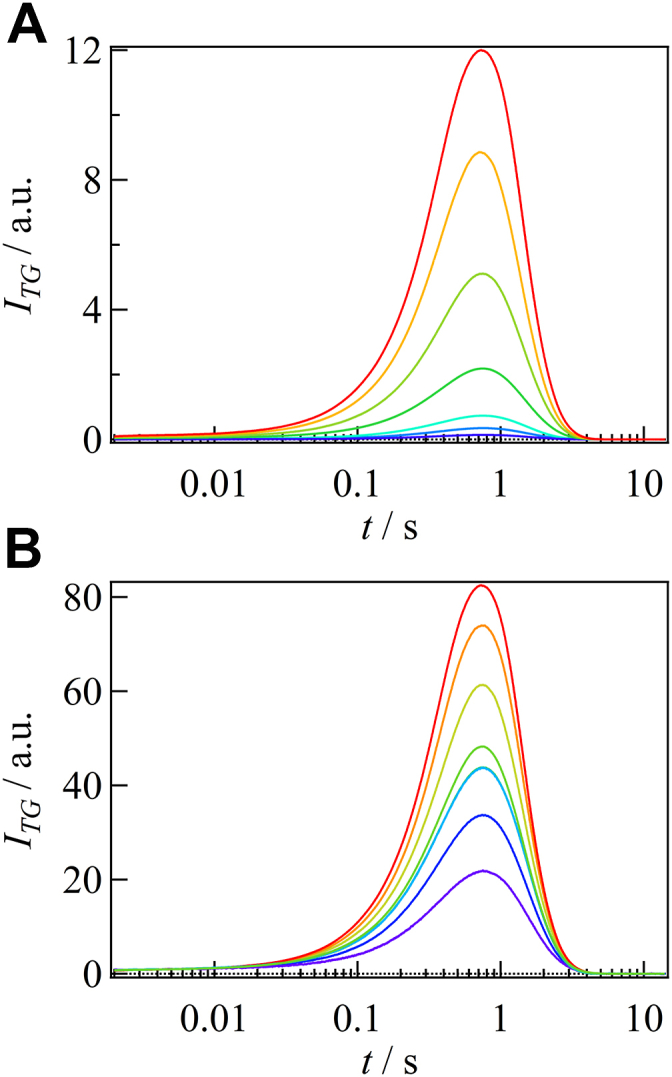
**Excitation light intensity dependence of the diffusion signal of FL-PAC.***A*, TG signal of FL-PAC obtained under various intensities of excitation pulse. The pulse intensities ranged from 0.69 to 135 μJ/pulse, representing a range from weak to strong signals. *B*, the signals are normalized by the intensity of species grating signal before the diffusion component. The diffusion signal intensity increases with increasing the light intensity and this result indicates that structural changes occur under strong light conditions. PAC, photoactivated adenylate cyclase; TG, transient grating.

Because FL-PAC exists as a dimer, the photoexcitation of one or two protomers in the dimer may induce different conformational changes, that is, the conformational changes of a dimer having one light-adapted and one dark-adapted protomers (LD-dimer) and two light-adapted protomers in the dimer (LL-dimer) could be different. To investigate this possibility, the dependence of the TG signal on the excitation light intensity was measured. Figure 3*A* displays the diffusion signals at *q*^2^ = 3.4 × 10^10^ m^–2^ at various excitation light intensities (6.9–135 μJ/pulse). If the conformational changes in the LD-dimers and LL-dimers were the same, the diffusion signal intensities would be the same for both samples. However, because the LL-dimer contains two excited protomers, the species grating signal intensity before the diffusion signal, which is proportional to the number of red-shifted molecules, should be twice that of the LD-dimer. Therefore, when the diffusion signal is normalized to the species grating signal intensity before the diffusion signal, the diffusion signal intensity should decrease with increasing the light intensity. Interestingly, even after such normalization, the intensity of the diffusion signal increased with increasing the light intensity (Fig. 3*B*). This result indicates that the LL-dimer undergoes a significantly larger *D*-change than LD-dimer. Furthermore, if the LD-dimer contributes to the diffusion signal, the shape of the diffusion signal is expected to change by changing the contribution of the LL-dimers. However, because the profile did not change with the light intensity, the contribution of the LD-dimer to the diffusion signal must be negligible. These findings suggest that the excitation of both protomers in the dimer is necessary to induce the *D*-change. Because the oligomeric form did not change upon light irradiation (SI-2), the observed *D*-change cannot be attributed to association/dissociation reactions. Instead, it is likely due to conformational changes in the protein moiety.

### CD spectroscopy

**Figure 4:**
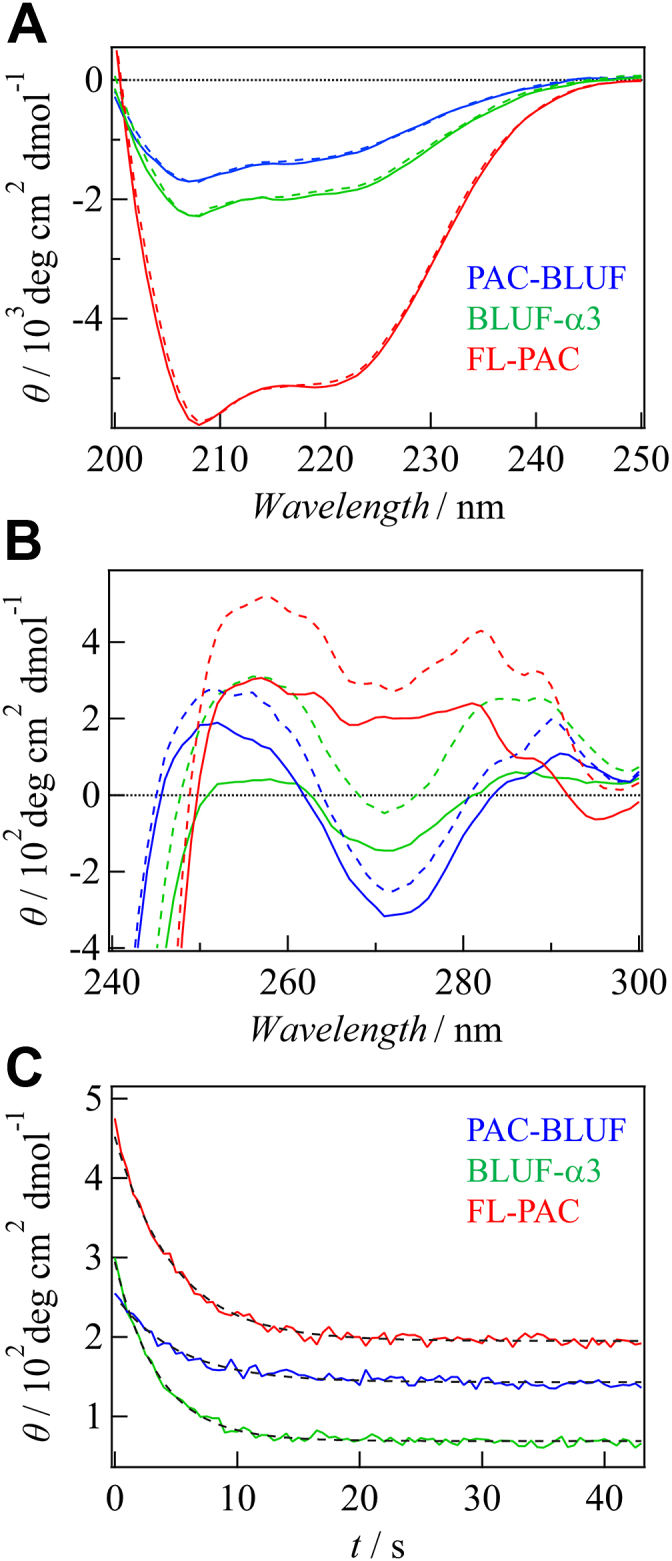
**CD spectra of FL-PAC, BLUF-α3, PAC-BLUF, and thermal recovery curves.***A*, Far-UV CD spectra of FL-PAC (*red*), BLUF-α3 (*green*), and PAC-BLUF (*blue*) obtained in the *dark* (*solid lines*) and *light* (*dashed lines*) states. The light-induced changes are very minor for all proteins. *B*, near-UV CD spectra of FL-PAC (*red*), BLUF-α3 (*green*), and PAC-BLUF (*blue*) obtained in the *dark* (*solid lines*) and *light* (*dashed lines*) states. *C*, thermal recovery of CD intensity at 258 nm for FL-PAC (*red*), BLUF-α3 (*green*), and PAC-BLUF (*blue*). The fitted curves using a single-exponential function for BLUF-α3 and PAC-BLUF and a double-exponential function for FL-PAC are shown with *dashed lines*. PAC, photoactivated adenylate cyclase.

To investigate the light-induced changes in the secondary structure, CD measurements were performed in the far-UV region (200–250 nm). Figure 4, *A* shows the CD spectra of the three constructs in the dark and light states. The intensity of the CD spectrum decreased as the domains were truncated, which is reasonable for the helical-rich structures of the α3 and AC domains. However, the spectra showed no significant differences between the dark and light states for any of the samples. Previously, using the same CD system, our group has detected clear light-dependent changes for various light sensor proteins (56, 57, 58). Compared with these previous results, the CD change of PAC by light irradiation is very small. Therefore, we consider that the secondary structural change of OaPAC is minor upon light illumination, at least within the sensitivity range of our CD measurement. Based on this measurement, we consider that the observed *D*-changes are not caused by changes in the secondary structure upon photoexcitation.

CD measurements were also performed in the near-UV region (250–300 nm) (Fig. 4*B*). Interestingly, light-induced changes were observed, and these changes exhibited the thermal recoveries in the dark after the light irradiation was stopped (Fig. 4*C*). The recovery rates agreed with those obtained from the absorption changes (SI-1), confirming that the CD changes were associated with the photocycling reaction. The observed near-UV CD spectra may contain contributions from flavin absorption. Nevertheless, the light-induced changes of the CD spectra of PAC-BLUF and the W90A mutant shown below are small, although the absorption spectral changes of flavin are almost the same as that of FL-PAC. Therefore, we consider that the observed CD changes are predominantly attributed to structural changes in the protein part.

Generally, the CD spectrum in this wavelength range is sensitive to the environment of aromatic amino acid side chains, including tryptophan, tyrosine, and phenylalanine, and it reflects the tertiary and quaternary structure of the protein (59, 60, 61). BLUF-α3 and FL-PAC exhibited more pronounced changes in the CD intensity upon light illumination than PAC-BLUF. This suggests that conformational changes occur not only in the BLUF domain but also in the α3-helix and AC domains. Mapping the distribution of the aromatic residues on the FL-PAC structure revealed their widespread distribution throughout the protein (Fig. S4), making it difficult to pinpoint specific structural changes detected by the CD measurements. Although a CD spectrum in this wavelength region can provide a useful fingerprint for protein identification and conformation, it is seldom used for direct structural analysis. However, the results indicate that the tertiary and/or quaternary structural changes occurred in the α3-helix and AC domains, which may be related to the *D*-change observed using the TG method.

### SAXS measurements

The reported crystal structures of OaPAC in the dark and light states showed minor changes in the protein structure, except around the chromophore (4). The small change might be caused by the suppression of possible movements due to the packing effect of the crystal field. For bPAC, the crystallographic structures revealed a slight opening motion in the AC domain upon light illumination (6). This finding led to a hypothesis that a similar opening movement may occur in OaPAC, which could be pronounced in the solution phase. If this is the case, it might explain the observed *D*-change. To test this hypothesis, the SAXS measurements were performed on FL-PAC.

**Figure 5:**
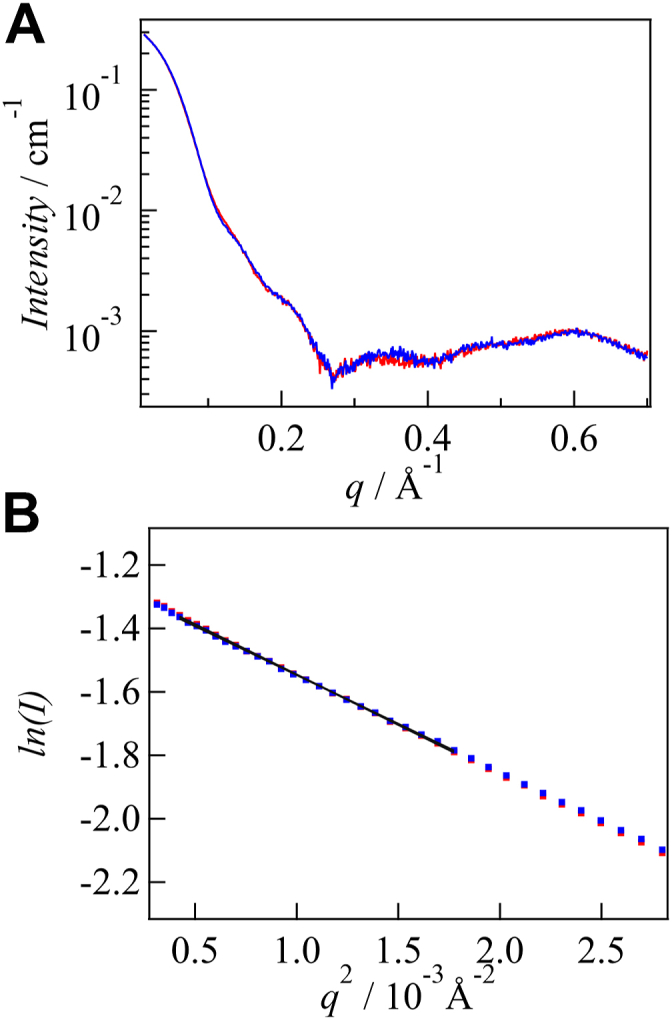
**Small angle X-ray scattering curve and Guinier plot of FL-PAC.***A*, SAXS profiles of FL-PAC obtained in the *dark* (*blue*) and *light* (*red*) states. *B*, Guinier plots, which show the dependence of the logarithm of the scattering intensity on the square of the scattering vector, for dark (*blue*) and light (*red*) states. The changes in the intensity and shape were very small upon light illumination. PAC, photoactivated adenylate cyclase; SAXS, small-angle X-ray scattering.

The SAXS curves obtained in the dark and light states are shown in Figure 5*A*. To measure the light state, complete photoconversion to the light state was confirmed by measuring the absorption spectrum (SI-5, Fig. S5*B*). No significant changes were observed in the SAXS curve upon light illumination, indicating that the overall shapes are very similar in the dark and light states. The curves calculated using the crystal structure reproduce the experimental curves well (SI-5, Fig. S5*D*). Hence, we conclude that the solution structures closely resemble the crystal structures in both the dark and light states. A slight difference between the calculated and experimental data may arise from the packing effect on the crystal structure and/or unresolved structure of the C-terminal end (amino acid residues G351-L366) of the crystallographic structure (3, 4).

Figure 5*B* presents the Guinier plots of *I*(*q*) in the low-angle region, which were used to estimate the molecular mass and radius of gyration (*R*_g_) (62, 63). The molecular mass was estimated to be 80 kDa for both the dark and light states, which is consistent with the dimeric form of OaPAC. Upon light illumination, *R*_g_ does not change within the range of measurement error (30 ± 1 Å for dark state and 31 ± 1 Å for light state). Additionally, maximum particle dimension (*D*_max_) was determined to be 88 ± 3 Å and 90 ± 3 Å in the dark and light states, respectively, by the analysis of the distance distribution function (SI-5). These analyses indicate no significant changes in the tertiary or quaternary structures. Therefore, the *D*-change detected using the TG method cannot be attributed to changes in the overall shape of the protein. The possible origin of the *D*-change is discussed later.

### Importance of the Trp residue

A previous study using TrA in the millisecond time range showed that the slowest component with a time constant of tens of milliseconds for OaPAC, SyPixD, and AppA disappeared when the conserved Trp residue was replaced with Ala (43). This result implied a significant role of the conserved Trp residue in the BLUF domains. To further investigate the importance of this residue in the conformational changes of the protein, the diffusion signal of the Trp mutant (W90A) of FL-PAC was examined. The absorption spectra in the dark and light states were similar to those of the WT protein, and the thermal recovery rate decreased (SI-6, Fig. S6).

**Figure 6:**
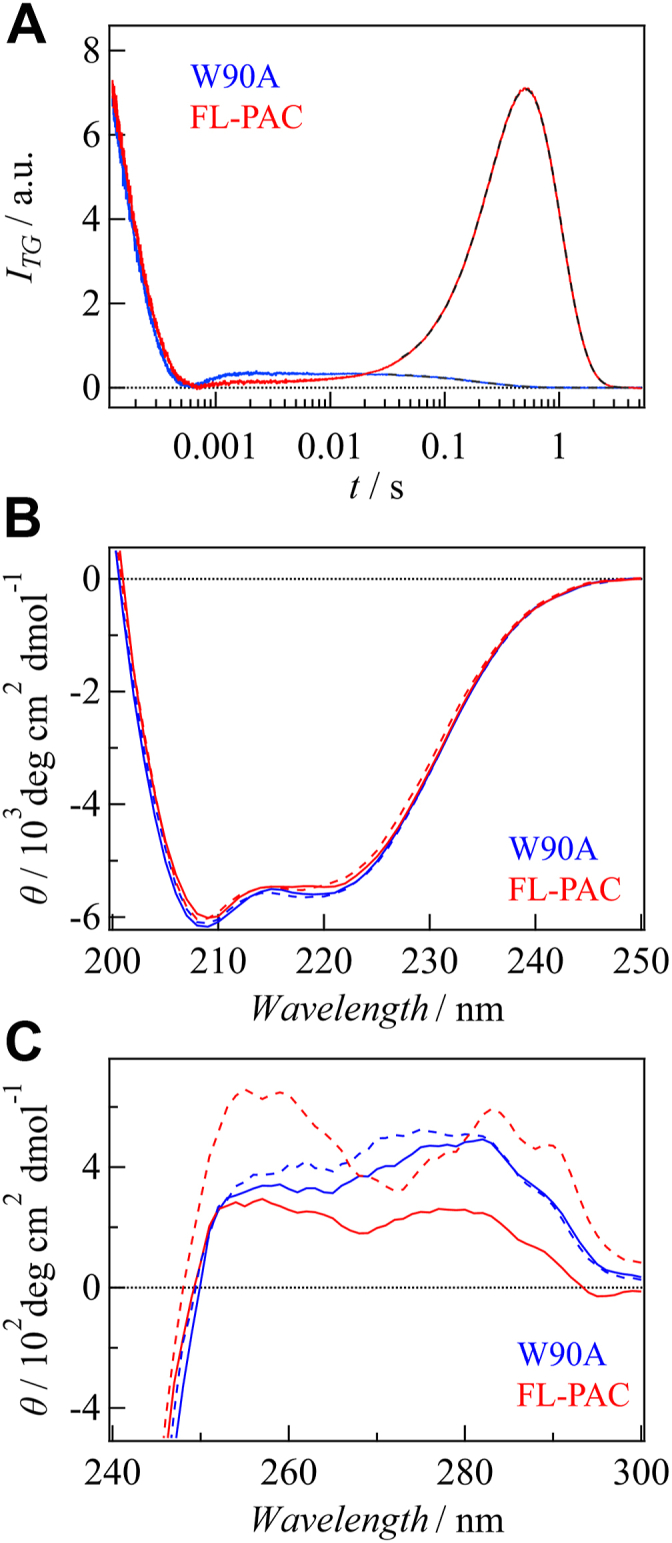
**TG and CD analyses of W90A mutant.***A*, TG signals of the W90A mutant (*blue*) and FL-PAC (*red*). *B*, far-UV CD spectra of the W90A mutant (*blue*) and FL-PAC (*red*) obtained in the *dark* (*solid lines*) and *light* (*dashed lines*) states. *C*, near-UV CD spectra of the W90A mutant (*blue*) and FL-PAC (*red*) obtained in the dark (*solid lines*) and light (*dashed lines*) states. These data represent that the light-induced structural changes of the W90A mutant are minor. PAC, photoactivated adenylate cyclase.

Figure 6*A* shows the TG signals of W90A and WT FL-PAC at *q*^*2*^ = 2.5 × 10^10^ m^–2^ and a concentration of 80 μM. Interestingly, the characteristic rise-decay signal observed for WT FL-PAC completely disappeared for the W90A mutant, indicating that the mutation suppressed DSCC. The monotonous decay signal was reproduced by considering a slight change in *D* upon photoexcitation (*D*_R_ = 6.2 × 10^11^ m^2^ s^–1^, *D*_P_ = 6.1 × 10^11^ m^2^ s^–1^). The *D*_R_ value is the same as that of the reactant of WT FL-PAC, suggesting that the structure in the dark state remains unaffected by the mutation, but the light-induced change in the protein structure was suppressed.

The effect of the mutation on the CD spectrum in the far-UV region was negligible for both dark and light states (Fig. 6*B*), suggesting that the secondary structure does not change. However, the CD spectra in the near-UV region showed differences (Fig. 6*C*). The spectrum of the dark state was altered by the mutation, particularly in the wavelength range of 260 to 300 nm, primarily because of the absence of the Trp residue. Notably, the light-induced change in the CD signal was significantly suppressed compared with that of the WT protein. Hence, these findings emphasize the crucial role of Trp in the transmission of light signals in FL-PAC.

### Catalytic activity

OaPAC exhibited significant conformational changes only when both protomers in the dimer were photoconverted to the light state. If this conformational change is relevant to the function of the protein, then the catalytic activity depended on the light intensity. To investigate the relationship between the conformational changes observed by the TG method and the function of OaPAC, the light intensity dependence of the catalytic activity was measured. The experimental scheme and detailed data analysis are presented in SI-7 (Fig. S7).

**Figure 7:**
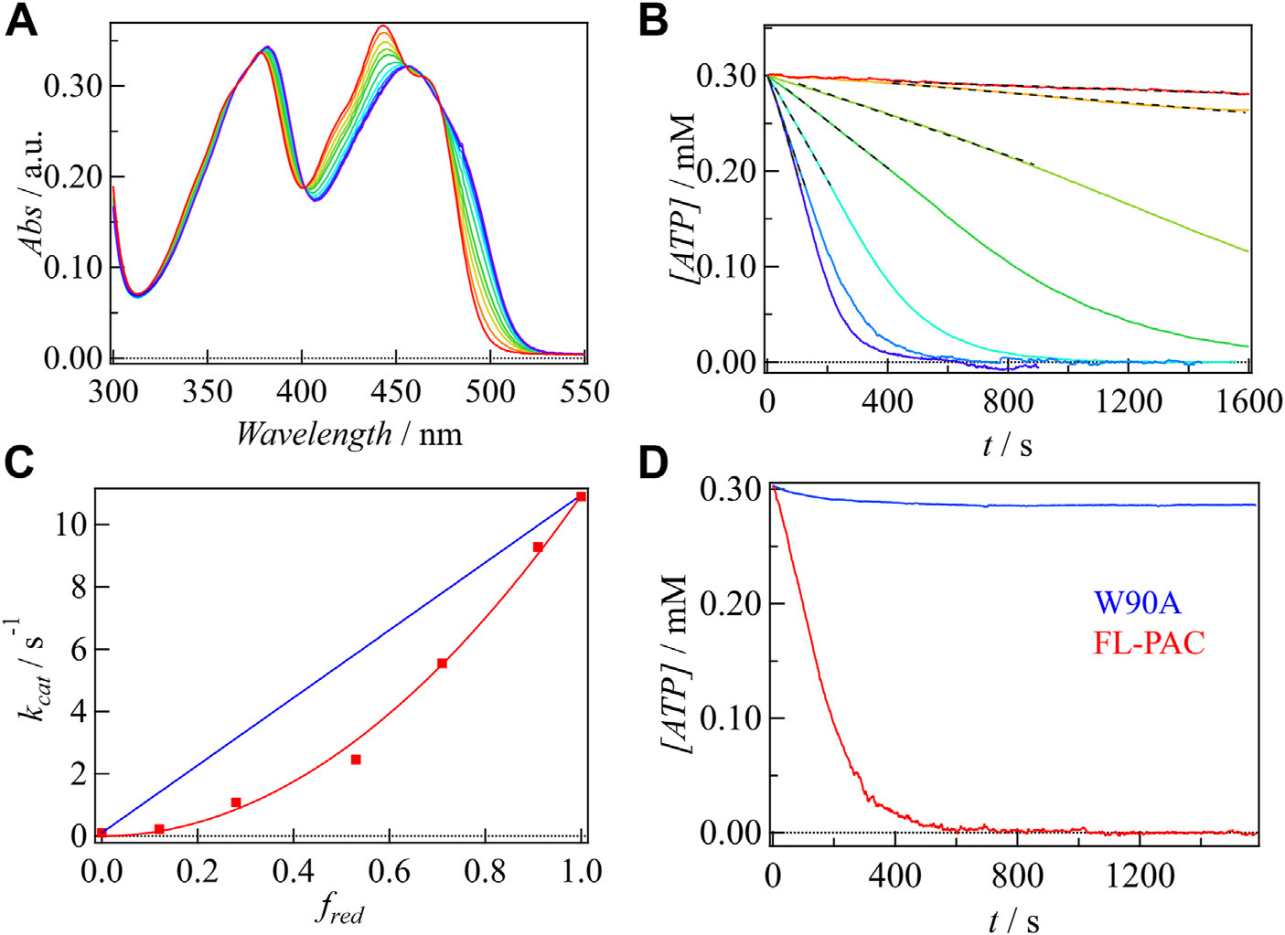
**Light intensity dependence and mutational effect on the enzyamatic activity of OaPAC.***A*, absorption spectra of FL-PAC at various intensities of light. *B*, the decay of ATP concentration in the presence of FL-PAC at various intensities of light. The linear ranges of the data were fitted by a linear function, shown by *black dashed lines*. *C*, plot of the catalytic rate constant (*k*_cat_) against the fraction of red-shifted species (*f*_red_). The data are fitted by a quadratic function (*red line*). A linear dependence is shown for comparison (*blue line*). *D*, the decay of ATP concentration in the presence of W90A (*blue*) and FL-PAC (*red*) in the light state. These data showed that the enzymatic activity of FL-PAC increases nonlinearly with light intensity and the W90A mutation impairs the enzyme activity. PAC, photoactivated adenylate cyclase.

The fraction of the light state (*f*_red_), which was monitored by the redshift in the absorption spectrum (SI-1), was controlled by adjusting the light intensity (Fig. 7*A*). For the measurement of the catalytic activity, we used MalionR, which is an ATP sensor protein with high sensitivity and specificity (64), within a linear detection range of 0 to 300 μM. Figure 7*B* shows the temporal change in the ATP concentration at various light exposures, and the catalytic rate constant (*k*_cat_) is plotted against *f*_red_ (Fig. 7*C*). The results show a quadratic increase in *k*_cat_ with respect to *f*_red_. This behavior indicates that a significant enhancement in the enzyme activity occurs only when both protomers in the dimer are photoconverted. Therefore, we conclude that DSCC is crucial for the enhancement of the catalytic activity. Additionally, we measured the activity of the W90A mutant and observed a loss of activity compared with that of the WT protein, as shown in Figure 7*D*. This result further confirms that DSCC is related to the function of OaPAC and highlights the critical role of the Trp residue in transmitting light signal.

## Discussion

The SEC experiments indicated that the oligomeric state of the protein does not change by light illumination. The CD measurements reveal no significant changes in the secondary structure of the protein. The previously reported crystallographic structures of OaPAC in the dark and light states showed that most light-induced conformational changes were limited to the chromophore region (3, 4). The SAXS curves in solution are consistently explained by the crystallographic structures, that is, there were no significant conformational changes in the global shape of the protein. Contrary to these observations, the significant light-induced conformational changes were detected in the TG measurements as DSCC. These findings imply that *D* is not only influenced by the size and shape of the molecule but also by its interactions with the surrounding solvent, including water molecules. Changes in the surface properties, such as the exposure of different numbers of the hydrophilic residues to the solvent upon light illumination, can result in detectable variations in *D*. These changes may be difficult to detect using other techniques.

It is worth noting that the BLUF domain generally possesses a rigid structure, and DSCCs have not been observed in other BLUF domains studied so far (54, 65). Despite the absence of DSCCs, the reaction still occurs within a millisecond timescale, which should be related to the signaling processes. Although PAC-BLUF does not show any *D*-change, BLUF-α3 exhibits a significant decrease in *D* with a time constant of 4.0 ms, followed by the slight increase in *D* with a time constant of 31 ms. Since CD measurements do not indicate any secondary structure change, the light-induced change could be a change in the orientation of the helix, such as rotation. The α3-helix has an amphipathic character (Fig. S8), containing both hydrophilic and hydrophobic residues, and forms a coiled-coil structure to stabilize the dimeric form (3, 4). If the rotational motion is induced by light, the exposed residues should change, which could be detected by the change in *D*. In the first step (4.0 ms), the hydrophilic residues may be exposed to the solvent, leading to an increase in the friction through intermolecular interactions with water molecules, which may result in a decrease in *D*. In the subsequent step (31 ms), the hydrophobic residues may be exposed or the hydrophilic residues may be buried by another rotational movement in the α3-helix to increase *D*. Because the rate of the first step is similar to that of the reaction phase of PAC-BLUF (2.8 ms), the movement of the α3-helix is induced by the local conformational change in PAC-BLUF, leading to another conformation with a time constant of 31 ms.

For FL-PAC, we observed a slight decrease in *D* at 2.3 ms, followed by a larger decrease at 36 ms. The similar rates of *D*-changes between the BLUF-α3 and FL-PAC suggest that DSCCs observed in FL-PAC are regulated by the motion of the α3-helix. The smaller *D*-change in the first step of FL-PAC compared to BLUF-α3 could be due to the presence of the AC domain, which partially restricts the rotational movement of α3. In the subsequent step, a notable decrease in *D* was observed for FL-PAC but not for BLUF-α3. This difference suggests that the AC domain undergoes significant DSCCs at this step (36 ms). It is reasonable to assume that the α3-helix regulates the change in the relative orientation of the AC domains upon light illumination. The interface between the AC domains in the dimer contains numerous hydrophobic and hydrophilic residues, suggesting that the rotational motion could influence *D*. Crystallographic data also revealed the presence of light-induced relative motions between BLUF and AC domains, characterized by a modest rotation of 1.3° and a slight screw translation of 0.13 Å (3, 4). Surprisingly, the very small movements were detected as DSCCs in a time-resolved manner. In a previous study using time-resolved infrared spectroscopy, light-induced changes in the IR spectrum were observed in FL-PAC within 3 ns upon photoexcitation (22). While the primary focus of the research was on studying ultrafast processes near the chromophore, the spectrum showed that the reaction was not complete even after 100 μs, particularly in amide bands (22). The structural changes observed in this study beyond milliseconds are likely to be associated with the changes in the IR spectrum.

Near-UV CD measurements also detected conformational changes. Several aromatic amino acids are located at the interface of the dimer and at the contact points between PAC-BLUF and the α3-helix, as well as at the connecting points between the α3-helix and the AC domain (Fig. S9). Hence, we suggest that the signal is transmitted from the PAC-BLUF domain to the AC domain *via* changes in the α3-helix. Indeed, substituting leucines on the α3 helices with alanine (L111A/L115A) has been reported to result in the loss of light-induced activation (3), underscoring the significance of hydrophobic interactions among the α3-helices. Moreover, at the junction between the α3 helices and the AC domains, Tyr-125 and Asn-256 form an intersubunit hydrogen bond that contributes to protein function (3). The signal transduction through the α3-helix is similar to that proposed for bPAC (6).

The importance of the C-terminal helices of the BLUF domains has been demonstrated in various BLUF proteins. For example, in PapB, unfolding of the C-terminal helix has been reported (57), and in the BLUF region of EB1, the C-terminal helix is essential for light-induced dimerization (55). In SyPixD, the C-terminal helices play a role in stabilizing the decameric structure in the dark and facilitating light-induced dissociation (66). Typically, the C-terminal helix forms hydrophobic interactions with the β-sheet of the BLUF domain. In the case of OaPAC, the C-terminal helix (α3-helix) also contributes to the dimer formation through the coiled-coil interactions (3). In this case, the PAC-BLUF domain regulates the activity of the C-terminal domain (AC domain) by altering the angle of the helix in a light-dependent manner, as described above. These findings highlight the diversity of signaling mechanisms among BLUF proteins.

Mutation of the Trp residue in the PAC-BLUF domain leads to the suppression of light-induced conformational changes. In the crystal structure, the side chain of Trp extends towards the N-terminal edge of the α3-helix (Fig. 1*A*), suggesting its potential role in transmitting signals from the PAC-BLUF domain to the helix (3, 4). The TG and CD analyses showed that the dark-state structure is unaffected by the Trp mutation, indicating that only light-dependent structural changes are inhibited. The TrA measurements revealed that the Trp mutant specifically abolishes the slowest reaction phase (36 ms), suggesting that this step is regulated by the Trp residue. Consequently, we propose that the initial step (2.3 ms) is governed by the changes in the interaction between the β-sheet of the BLUF core and the α3-helix, while the subsequent step (36 ms) is regulated by the motion of the Trp residue. At this step, the AC domain underwent a conformational change, as indicated by a decrease in *D*. It has also been suggested that a slight alteration in the conformation of the Trp influences the hydrogen bonding arrangement with the neighboring residues such as Arg-106, which is located on the α3-helix of the partner protein in the dimer (4). The involvement of Trp in signal transmission has been proposed in other BLUF proteins as well (43, 67, 68, 69, 70, 71), and although there may be variation in the structure and orientation of the C-terminal helix, signal transmission mediated by Trp is considered a common mechanism among the BLUF proteins.

Interestingly, DSCCs in FL-PAC occurred specifically when both protomers in the dimer were photoexcited (LL dimer). This finding suggests that the torque force generated by the photoconversion of one protomer is insufficient to induce the rotational motion of the AC domain. It is proposed that light information is transmitted from both BLUF domains to the helix, resulting in activation of the AC domain. Furthermore, since the enzymatic activity of the LL-dimer is much higher than that of the LD-dimer, it is reasonable to consider that DSCCs play a crucial role in enhancing enzymatic activity and that OaPAC functions as a nonlinear light intensity sensor that controls the concentration of cAMP only under strong light.

PACs have the ability to activate cAMP-dependent pathways, enabling light control over various biological responses. A common issue in optogenetic applications is the leakage of enzyme activity in the dark or under low-light conditions. Since the activity of OaPAC in the dark state is the lowest among the PAC proteins studied so far and it functions only under strong light condition, it enables more precise light control without any activity leakage under low-light conditions. Our finding here indicates that OaPAC is an ideal system for a tool of optogenetics.

Among enzyme-type BLUF proteins, BlrP1 consists of a BLUF domain and an EAL domain, which has a function of a phosphodiesterase to hydrolyze the bacterial second messenger c-di-GMP (72). While BlrP1 also forms a dimer, the orientation of the α3 helix and the relative arrangement of domains are different from those of OaPAC (72). In the reaction of BlrP1, the light-dependent structural change is observed in the enzymatic EAL domain, which is attributed to a clamshell-like opening motion of EAL dimers (54, 73), and these features are different from OaPAC's mechanism. Hence, even within the same enzyme-type BLUF proteins, diverse activation mechanisms exist depending on their different functions.

Many BLUF proteins, such as SyPixD, TePixD, and Blrp1, form oligomers, and it was found that the photoreactions of these proteins and OaPAC in this study depend on the intensity of excitation light (54, 74, 75, 76). For example, the dissociation reaction of SyPixD occurs only when multiple protomers are excited simultaneously (75, 76). The biological function of Blrp1 (catalyzing hydrolysis of c-di-GMP) is induced upon two protomer excitation in the dimer (77). Through the utilization of various oligomeric forms, it is suggested that numerous BLUF proteins have the function of nonlinear light intensity sensors.

### Concluding remarks

In this study, the photoreaction dynamics of OaPAC were investigated mainly using the TG, CD, and SAXS techniques. Although no significant changes were observed in the oligomer state (SEC), secondary structure (CD), or overall molecular shape (SAXS), light-induced structural changes were successfully detected by the TG method as the diffusion change in a time-resolved manner. By analyzing the truncated mutants, we uncovered the transmission route of light information from the PAC-BLUF domain to the AC domain through the α3-helix, with the highly conserved Trp residue in the PAC-BLUF domain, which plays a critical role in signal transmission. Additionally, we discovered that these structural changes occur only when both monomers in the dimer undergo photoconversion, and we further demonstrated that the enzymatic reaction of the AC domain is indeed activated in the LL-dimer. Based on these results, we suggest that OaPAC functions as a nonlinear light intensity sensor.

**Figure 8:**
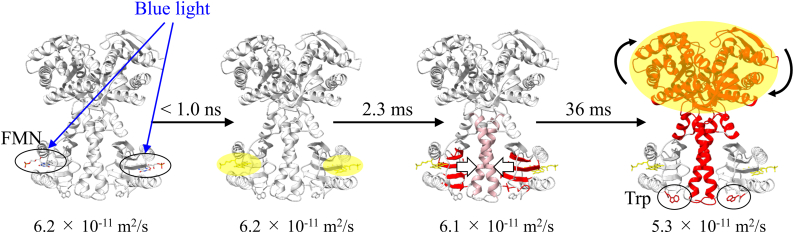
**Proposed reaction scheme of OaPAC.** The transmissions of the light signal from the chromophore to the AC domain are depicted as color changes. Alterations in the hydrogen-bonding network near the chromophore occur within 1 ns, followed by signal propagation from the PAC-BLUF domain to the α3-helix, inducing subtle structural changes. In the subsequent step, the α3-helix undergoes conformational changes facilitated by Trp, leading to an alteration in the relative orientation of the AC domains for functional activation. The final step is relevant to enhancing catalytic activity in the AC domain, occurring only when both protomers in the dimer are photoconverted, suggesting that OaPAC functions as a nonlinear light intensity sensor. The diffusion coefficients of each species are also shown. AC, adenylate cyclase; OaPAC, PAC from Oscillatoria acuminata; PAC, photoactivated adenylate cyclase.

The reaction scheme of OaPAC elucidated by this study is shown in Figure 8. Upon photoexcitation of the chromophore, the hydrogen-bonding network surrounding the chromophore changes within 1 ns. The local conformational changes are transmitted to the α3-helix through the alteration in the interaction between the β-sheet of the BLUF core and the α3-helix, with a time constant of 2.3 ms. Subsequently, the α3-helix undergoes rotational motion driven by the conformational change of the Trp residue, resulting in a change in the relative orientation of the AC domains. This conformational change occurs with a time constant of 36 ms and is observed only when both protomers in the dimer undergo photoconversion. This final step is relevant to the enhancement of the catalytic activity in the AC domain.

The nonlinear light intensity response could be important for optogenetics by enabling precise light manipulation without any activity leakage at low-light intensities. BLUF proteins often form oligomers, and some of them exhibit light intensity–dependent photoreactions and activities. For example, similar nonlinear response was found for BlrP1 (54, 77). Consequently, we consider that a significant number of BLUF proteins function as nonlinear light intensity sensors by forming oligomers.

## Experimental procedures

### Sample preparation

To express OaPAC, a customized pET 2M-T vector (RRID: Addgene_29708; a gift from Scott Gradia) was used. This vector originally contained a maltose-binding protein tag and a tag cleavage sequence for the tobacco etch virus protease. In this study, these sequences were replaced with recognition sequences for the human rhinovirus (HRV) 3C protease. Two truncated mutants, PAC-BLUF and BLUF-α3, as well as a mutant in which Trp90 was replaced with Ala (W90A) were prepared using FL-PAC as a template and appropriate primer sets through the standard PCR method.

These expression vectors were transformed into *Escherichia coli* BL21(DE3) cells and cultured in LB medium at 37 °C until the OD600 reached 0.6. IPTG was then added to the medium to a final concentration of 0.1 mM, and the cells were incubated at 18 °C for approximately 20 h. Subsequently, the cells were harvested by centrifugation at 4000×*g* for 15 min at 4 °C and suspended in PBS (pH 7.5) containing DNaseI and an excess amount of FMN. After cell lysis by sonication, the homogenate was centrifuged at 20,000*g* for 1 h at 4 °C. The protein was purified from the supernatant by Ni-affinity column chromatography (HisTrap HP, Cytiva). Following the exchange of the medium with PBS (pH 7.5) using a desalting column, the N-terminal His-tag was cleaved using the HRV 3C protease. The cleaved His-tag polypeptide and the uncleaved fusion protein were trapped by passing them over a Ni column. The flow-through from the column was collected and further purified by SEC using the Superdex 200 Increase 10/300 (Cytiva). The purities of the obtained proteins, determined by SDS-PAGE, were confirmed to be > 98%. The protein concentration was calculated based on the FMN molar absorption coefficient at 473 nm of ε_473_ = 9200 M^–1^ cm^–1^ prior to use.

### TG measurements

The experimental setup for the TG measurements followed previously reported methods (48, 51, 52, 53). The sample solution was excited using a XeCl excimer laser (308 nm, Compex102, Lambda Physik)-pumped dye laser (462 nm, HyperDye 300, Lumonics). A He-Ne laser (633 nm, 1144p, JDS Uniphase) was used as the probe laser. The grating wavenumber (*q*) was determined by measuring the decay rate of the thermal grating signal of bromocresol purple in an aqueous solution. The repetition rate was set to 0.04 Hz to avoid excitation of the light-adapted state. Approximately 20 signals were averaged using a digital oscilloscope to improve signal to noise ratio. The intensity of the laser pulse was measured using a joulemeter (J3-09, Coherent). The experiments were conducted at a temperature of 23 °C, and the sample concentration was set to 80 μM. TG measurements were conducted on three batches of protein samples independently purified, and uncertainties of fitting parameters were evaluated from these data.

The principle of the TG method has been described previously (47, 48, 51, 53). Briefly, there are two dominant contributions in the TG signal under the present experimental conditions: the thermal grating due to the thermal energy from photoexcited molecules and the species grating component due to the depletion of the reactant and the creation of the product including any transient species. The TG signals are expressed by the following function:

(1) $$I_{\text{TG}}\left(t\right)=\alpha {\left\{\delta n_{\text{th}}\;\exp\left(-D_{\text{th}}q^{2}t\right)+\delta n_{\text{spe}}\left(t\right)\right\}}^{2}$$

where α is a constant representing the system sensitivity, *δn*_th_ is the pre-exponential factor of the thermal grating signal, and *D*_th_ is the thermal diffusivity of the solvent. The fitting function of *δn*_spe_(*t*) depends on the reaction model. If *D* changes within the observation time window, the time profile should be analyzed by considering this change. For Reaction scheme 1, which included an intermediate species (I) before the final product, the time dependence of *δn*_spe_(*t*) is given by (48):

(2) $$\delta n_{\text{spe}}\left(t\right)=-\delta n_{R}\;\exp\left(-D_{R}q^{2}t\right)+\delta n_{I}\;\exp\left\{-\left(D_{I}q^{2}+k\right)t\right\}+\delta n_{P}\left[k\;/\left\{\left(D_{P}-D_{I}\right)q^{2}-k\right\}\right][\exp\left\{-\left(D_{I}q^{2}+k\right)t\right\}-\exp\left(-D_{P}q^{2}t\right)]$$

where *δn*_i_ and *D*_i_ (i = R, I, and P) represent the refractive index change and *D* of the corresponding species, respectively. For Reaction scheme 2, which involves two intermediate species (I_1_ and I_2_) before the final product, the time dependence of *δn*_spe_(*t*) is given by (76):

(3) $$\delta n_{spe}\left(t\right)=-\delta n_{R}\;\exp\left(-D_{R}q^{2}t\right)+[\delta n_{I1}-\delta n_{I2}\left\{k_{2}\;/\;\left(k_{2}-k_{3}\right)\right\}+\delta n_{P}\left\{k_{2}k_{3}/\;\left(k_{2}-k_{3}\right)\right\}\left\{1\;/\;\left(D_{I1}-D_{P}\right)q^{2}+k_{2}\right\}]\exp\left\{\left(-D_{I1}q^{2}+k_{2}\right)t\right\}+\left[\delta n_{I2}\left\{k_{2}\;/\;\left(k_{2}-k_{3}\right)\right\}-\delta n_{P}\left\{k_{2}k_{3}\;/\;\left(k_{2}-k_{3}\right)\right\}\left\{1\;/\;\left(D_{I2}-D_{P}\right)q^{2}+k_{3}\right\}\right]\exp\left\{\left(-D_{I2}q^{2}+k_{3}\right)t\right\}+\delta n_{P}\left\{k_{2}k_{3}\;/\;\left(k_{2}-k_{3}\right)\right\}\left[\left\{1\;/\;\left(D_{I2}-D_{P}\right)q^{2}+k_{3}\right\}-\left\{1\;/\;\left(D_{I1}-D_{P}\right)q^{2}+k_{2}\right\}\right]\exp\left(-D_{P}q^{2}t\right)$$

where *δn*_i_ and *D*_i_ (i = R, I1, I2, and P) represent the refractive index change and *D* of the corresponding species, respectively. By analyzing the time-dependent diffusion signals using these fitting functions, it is possible to determine the *D* values for each species as well as the rate constants associated with the *D*-changes.

(Scheme 1) $$R\overset{h\nu }{\to }I\overset{k_{1}}{\to }P$$

### Absorption measurements

For absorption measurements, a diode array spectrophotometer (Agilent Cary 8454, Agilent Technologies) was used. The measurements were performed in a quartz cell with an optical path length of 1.0 cm, and the sample concentration was 50 μM. To obtain absorption spectra in the light state, the sample solutions were exposed to illumination from a blue LED (480 nm, CHR-3S, Nissin Electronic Co) positioned above the sample cell. To observe the thermal recovery process of the light-adapted state, the sample solutions were initially illuminated with the blue LED for 20 s before the measurements. The thermal recovery was monitored at 495 nm. The temperature was maintained at 23 °C throughout the measurements.

### SEC measurements

The oligomeric state of the proteins in solution was determined using SEC with a Superdex 200 Increase 10/300 column (Cytiva). The column was pre-equilibrated with PBS buffer and maintained at room temperature. The sample concentration was set to 50 μM, and a volume of 100 μl was injected for each measurement. For the light state measurement, the column was irradiated with a Xe lamp (Max-302, Asahi Spectra). To calibrate the molecular mass based on the peak position, a gel filtration standard from Merck was utilized.

### CD measurements

CD spectra were obtained using a J-729W1 spectrometer (Jasco). To minimize interference from oxygen, the sample chamber was continuously purged with nitrogen gas at a flow rate of 15 L/min. The background signal from the buffer solution was subtracted from all measurements. The sample cell had an optical path length of 1.0 cm, and the protein concentration was 0.5 μM for far-UV measurements and 10 μM for near-UV measurements. To measure the CD spectrum in the light-adapted state, blue light from the LED (480 nm) was applied during the CD measurement. Since the detector used for CD measurement was sensitive only to UV light, the blue light illumination did not interfere with the measurement. To observe the thermal recovery process of the light-adapted state, the sample solutions were illuminated with the blue LED for 20 s prior to the measurement.

### SAXS measurements

The SAXS data were collected with the Photon Factory beamline BL-15A2. The sample solution was allowed to flow at a rate of 0.02 ml/min to minimize the radiation damage. To obtain the scattering curve in the light-adapted state, the sample solution was illuminated using a diode laser (450 nm, L450P1600MM; Thorlabs). Scattering intensities were measured using a PILATUS2M detector (DECTRIS) positioned at a distance of 1000 mm from the sample cell, with an exposure time of 200 s. One-dimensional scattering data, *I*(*q*), as a function of *q* (*q* = 4πsinθ/λ, where 2θ is the scattering angle and λ is the X-ray wavelength of 1.0 Å), were obtained by radial averaging of the measured scattering intensities. To obtain the scattering intensities on an absolute scale, the measured scattering intensities were calibrated based on the scattering intensity of water. Data processing was performed using SAngler and software within the ATSAS package (78, 79, 80). Experimental *I*(*q*) data in the *q*-range of 0.008 to 0.750 Å^–1^ were used for subsequent analyses. The radius of gyration (*R*_g_) and forward-scattering intensity (*I*(0)) were estimated from the Guinier plot of *I*(*q*) in the low-angle region. The theoretical *I*(*q*) of the model structure was calculated using WAXSIS (81). The distance distribution function (*P*(*r*)) was calculated using GNOM (82). The maximum particle dimension (*D*_max_) was estimated from the *P*(*r*) function as the distance (*r*) at which *P*(*r*) = 0. To simultaneously acquire the absorption spectrum of the sample during the SAXS measurements, white light (L10290, Hamamatsu) was focused using a collimating lens and directed to the position where the X-rays passed through the sample. The transmitted light was collected using an optical fiber and sent to a spectrometer (Flame, Ocean Insight) to obtain the absorption spectrum. This enabled the determination of the actual accumulation ratio of the light-adapted state during measurements under light illumination. SAXS measurements were conducted on three batches of protein samples independently purified, and uncertainties of fitting parameters were evaluated from these data.

### Enzymatic assay

In the enzymatic assay, the substrate ATP (Sigma–Aldrich) was incubated with FL-PAC, and the decrease in ATP concentration was monitored using an ATP sensor called MaLionR (64). MaLionR is an artificial fluorescent protein that incorporates the ϵ subunit of the ATP-binding region from bacterial F_0_F_1_-ATP synthase into the red fluorescent mApple protein (64). The ATP-dependent conformational change of the ϵ subunit, triggered by ATP binding, leads to changes in the absorbance and fluorescence of the mApple region. Therefore, the ATP concentration in solution can be quantified by measuring the changes in absorbance and/or fluorescence. In this study, changes in absorbance rather than changes in fluorescence was measured because the sample was illuminated with various intensities of blue LEDs to excite FL-PAC, which affects the fluorescence of MaLionR.

To investigate the relationship between the catalytic rate constant (*k*_cat_) and fraction of the red-shifted state (*f*_red_), the intensity of the blue LED was controlled using optical filters. First, the absorbance of the FL-PAC solution was measured at 495 nm using a UV-Vis spectrophotometer (Agilent 8454; Agilent Technologies) in the absence of ATP and MaLionR. The value of *f*_red_ was calculated for each illumination intensity using the following equation:

(1) $$f_{\text{red}}=\left(A_{495}\left(I\right)-{A_{495}}_{,D}\right)\;/\;\left(A_{495,L}-A_{495,D}\right)$$

where A_495,D_, A_495,L_, and A_495_(I) represent the absorbance at 495 nm in the dark state, fully red-shifted state, and tested sample, respectively.

Subsequently, a mixture of Mg-ATP and MaLionR was added to the OaPAC solution to initiate the enzymatic reaction. The intensity of the illumination light was maintained to keep the *f*_red_ constant during measurement. The concentrations of OaPAC and MaLionR were fixed at 15 μM and 20 μM, respectively, and the initial ATP concentration was set to 300 μM. ATP consumption was observed as a time-dependent change in the absorbance at 570 nm. The catalytic rate constant (*k*_cat_) was calculated based on the velocity obtained within the linear range without considering the Michaelis constant (*K*_M_). All assays were performed at 23 °C in PBS buffer. Details of the analyses are described in Section SI-7.

## Data availability

Data are available in the supporting information. All remaining data are contained in the article.

## Supporting information

This article contains supporting information.

## Conflict of interest

The authors declare that they have no conflicts of interest with the contents of this article.
