# Supplementary material for: Time-resolved study on signaling pathway of photoactivated adenylate cyclase and its nonlinear optical response
Source: J Biol Chem. 2023 Sep 22;299(11):105285. doi: 10.1016/j.jbc.2023.105285 (PMC10634658; doi:10.1016/j.jbc.2023.105285)
Supplement: Supplemental information [file mmc1.docx]

**Supporting information**

**Time-resolved study on signaling pathway of photoactivated adenylate cyclase and its nonlinear optical response**

Yusuke Nakasone ^a^, Hiroto Murakami ^a^, Shunrou Tokonami ^a^, Takashi Oda ^b^, Masahide Terazima ^a^

*^a^ Department of Chemistry, Graduate School of Science, Kyoto University, Kyoto 606-8502, Japan*

*^b^Department of Life Science and Research Center for Life Science, College of Science, Rikkyo University, Tokyo 171-8501, Japan*

**SI-1.** **Absorption spectra and thermal recovery**


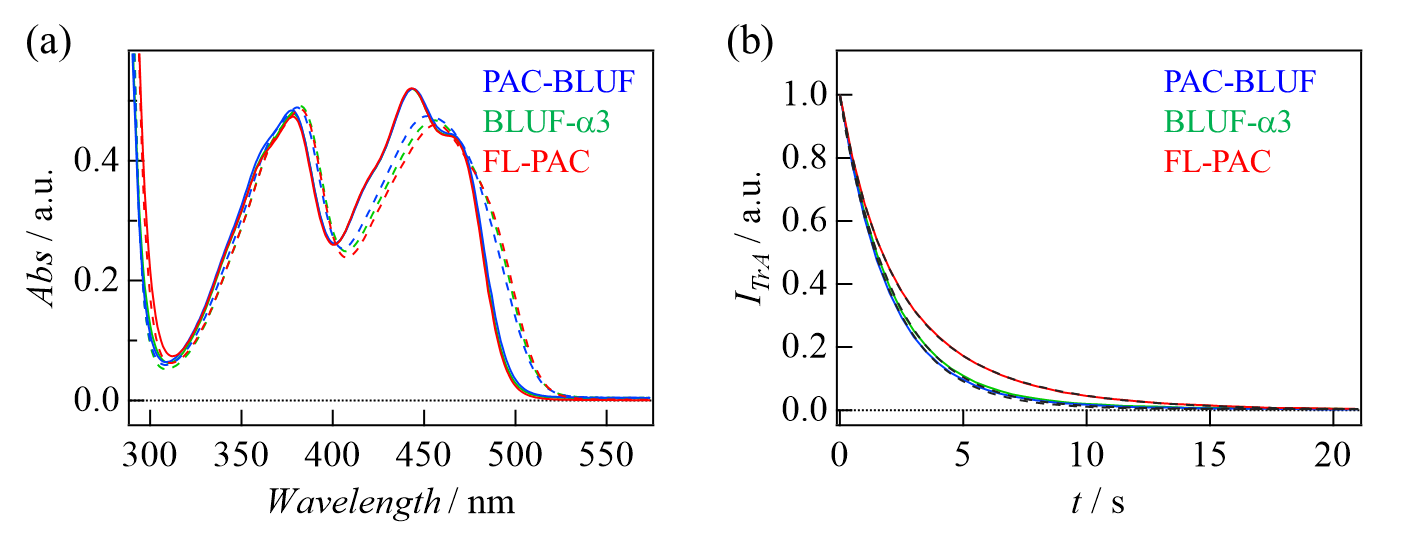


Figure S1. (a) Absorption spectra of FL-PAC (red), BLUF-α3 (green), and PAC-BLUF (blue) in the dark (solid lines) and light (broken lines) states. (b) Thermal recoveries of absorption at 495 nm for FL-PAC (red), BLUF-α3 (green), and PAC-BLUF (blue). The fitted curves using a single exponential function for BLUF-α3 and PAC-BLUF, and a double-exponential function for FL-PAC are shown with dashed lines.

**SI-2.** **Size exclusion chromatography**


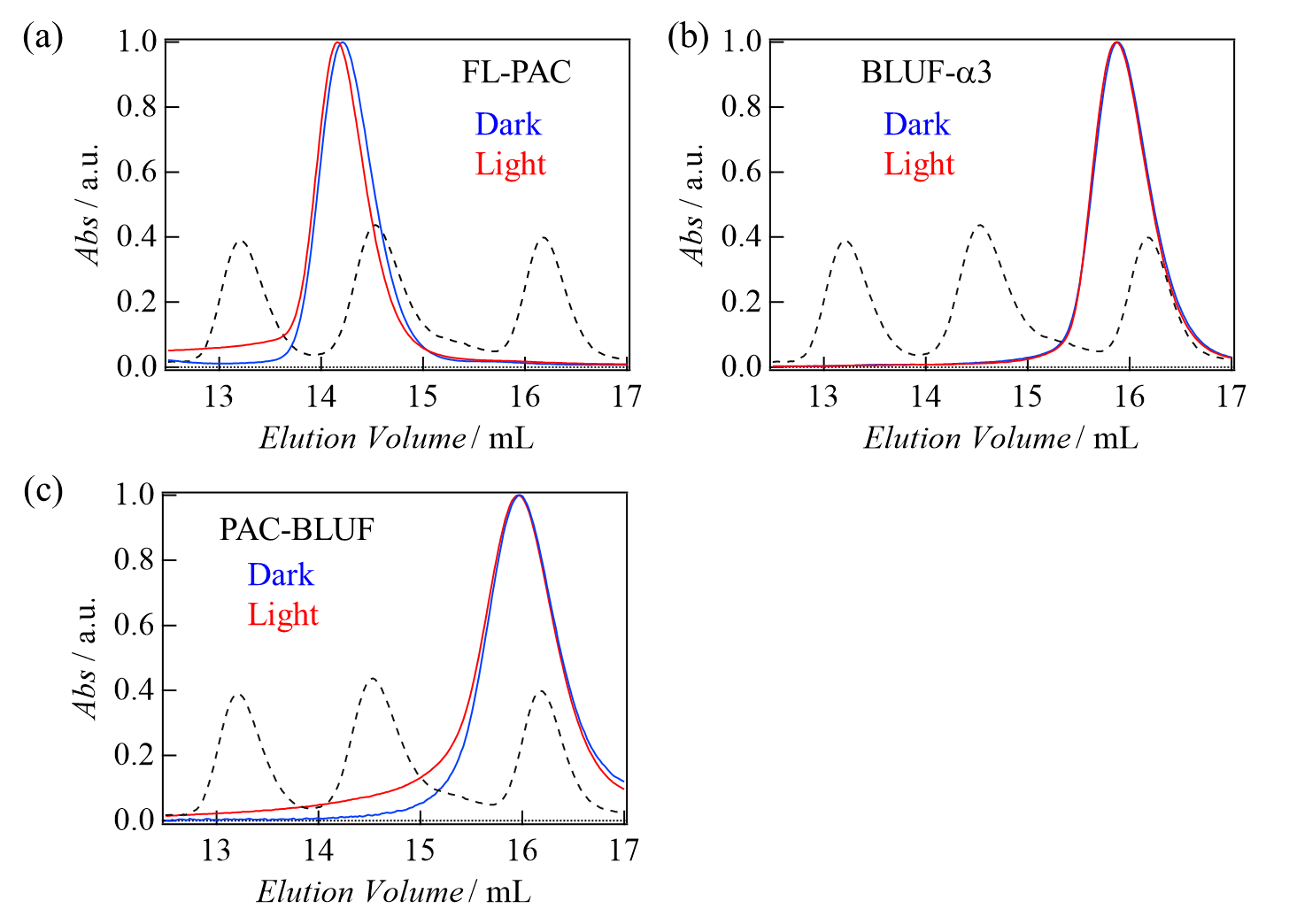


Figure S2. SEC profiles of (a) FL-PAC, (b) BLUF-α3, and (c) PAC-BLUF obtained in the dark (blue solid lines) and light (red solid lines) states. The profiles are normalized by the peak intensities to show changes in the peak positions clearly. The elution profiles of marker proteins are also shown with black broken lines. The three peaks observed in the figures correspond to alcohol dehydrogenase (150 kDa), bovine serum albumin (66 kDa), and carbonic anhydrase (29 kDa) from left to right. The calculated molecular masses from the elution profiles are 79 kDa for FL-PAC, 34 kDa for BLUF-α3, and 32 kDa for PAC-BLUF.

**SI-3. Global analyses of diffusion signal of FL-PAC**

In the main text, the *q*-dependence of TG signal of FL-PAC was analyzed based on Scheme 2 and revealed a slight decrease in *D* during the initial step, followed by a more pronounced decrease. However, the change in *D* during the initial step is small. To confirm the presence of *D*-change at the initial step, we analyzed the signals assuming no *D* change (*D*_I1_ = *D*_I2_) during the transition from I_1_ to I_2_ state in scheme 2. The systematic and relatively large deviations of the residues shown below indicates that the signal was not reproduced well using this assumption (Fig. S3(b)). Hence, the small change in *D* indeed occurs at the initial step.


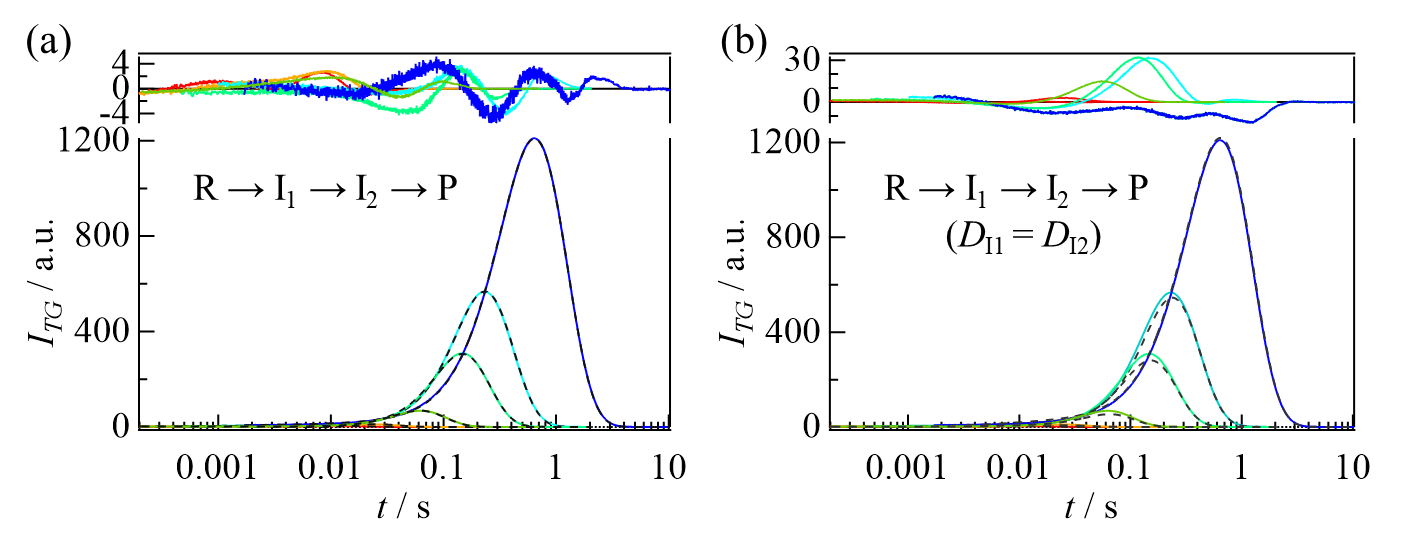


Figure S3. The *q*-dependence of diffusion signal of FL-PAC (Figure 2d in the main text) and the best-fitted curves with residues. The fitted curves were calculated based on Scheme 2 with the rate constants (2.3 ms)^–1^ and (36 ms)^–1^ with (a) *D*s listed in Table 1, and (b) by assuming *D*_I1_ = *D*_I2_. The profiles of the residues indicate that *D*_I1_ and *D*_I2_ are slightly different.

**SI-4.** **Mapping of aromatic residues on crystal structure of OaPAC**


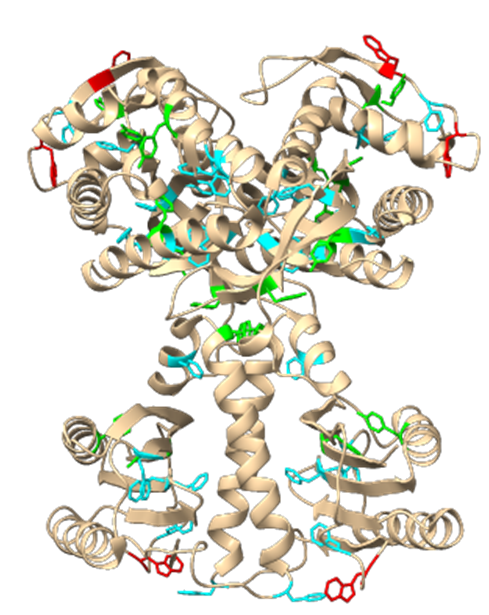


Figure S4. (a) Mapping of the aromatic residues on the crystal structure of OaPAC (PDB ID: 4yut). Tryptophan, tyrosine, and phenylalanine are shown in red, green, and blue, respectively.

**SI-5.** **SAXS measurements and analyses**


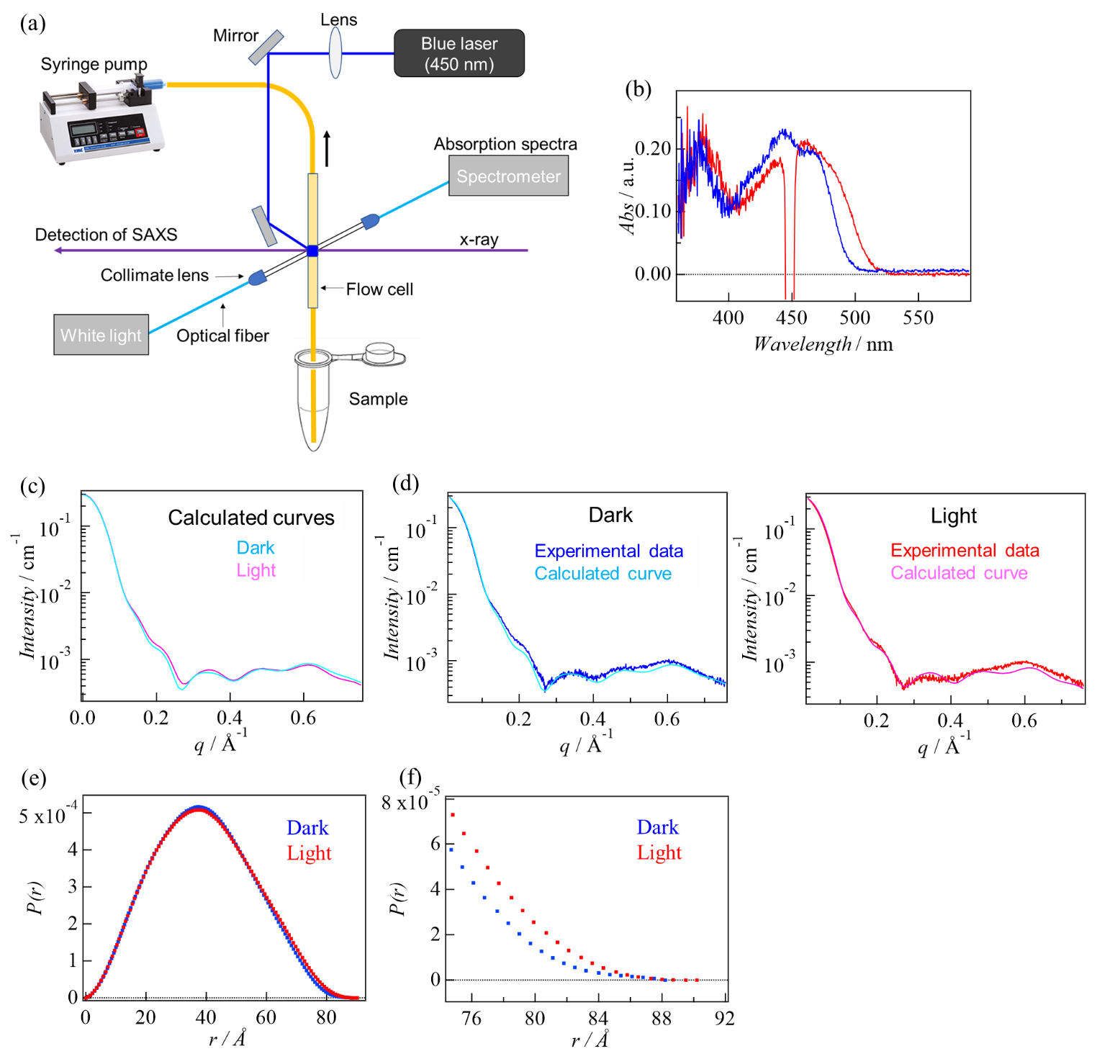


Figure S5. (a) Experimental setup for SAXS measurements. The sample solution was flowed at a rate of 0.02 mL/min to minimize radiation damage. To obtain the scattering curve in the light-adapted state, the sample solution was illuminated using a diode laser (450 nm). To simultaneously acquire the absorption spectrum of the sample during SAXS measurements, white light was focused using a collimating lens and directed to the position where the X-rays passed through the sample. The transmitted light was collected using an optical fiber and sent to a spectrometer to obtain the absorption spectrum. (b) Absorption spectra obtained during the SAXS experiment for the dark (blue) and light (red) states. The full photoconversion was confirmed by the change in the absorption spectrum (red-shift). The negative spike observed at the wavelength of 450 nm is due to scattering light from the excitation laser. (c) SAXS curves calculated from crystal structures of OaPAC for both the dark (cyan) and light (purple) states. WAXSIS software (http://waxsis.uni-goettingen.de/) was used for the calculation of the solution scattering. The crystal structures of OaPAC in the dark (PDB ID: 4yut) and light (PDB ID: 5x4u) states were used as atomic structures for the calculation. (d) Comparison of experimental data with calculated curves. (e) The distance distribution function (P(r)), which was calculated using GNOM (https://www.embl-hamburg.de/biosaxs/gnom.html), is shown for both the dark and light states. The maximum particle dimension (*D*_max_) was estimated from the *P*(*r*) function as the distance (*r*) at which *P*(*r*) = 0. The curves at the long distance region of the *P*(r) function are magnified in (f).

**SI-6.** **Absorption spectra and thermal recovery of W90A mutant**


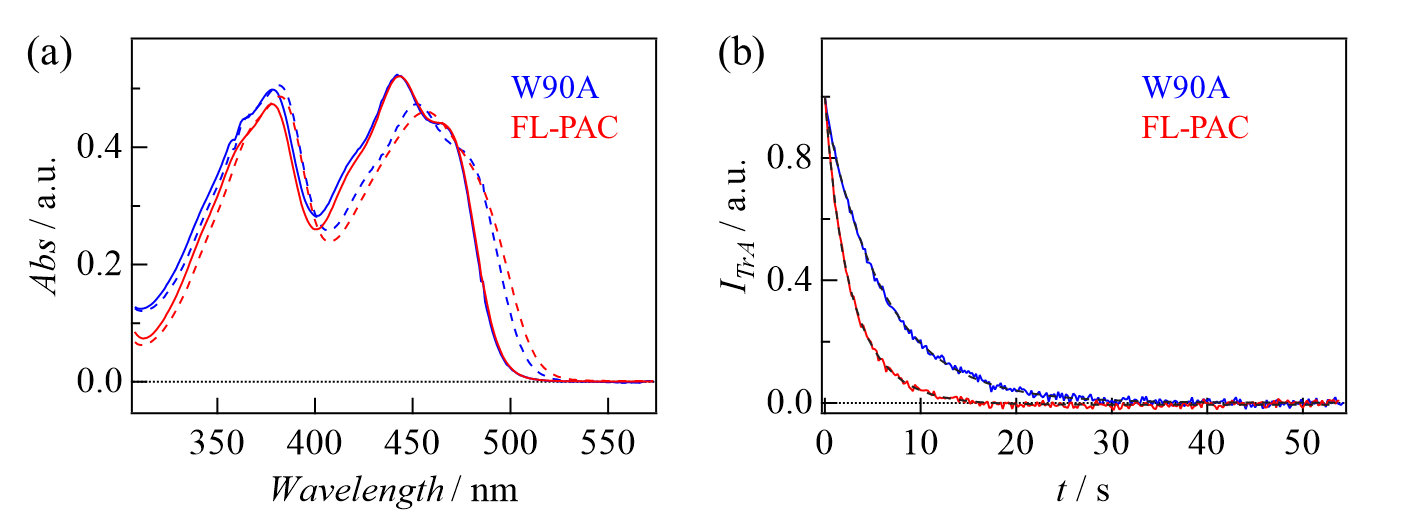


Figure S6. (a) Absorption spectra of W90A (blue) and wild-type (red) FL-PAC in the dark (solid lines) and light (broken lines) states. (b) Thermal recoveries of absorption at 495 nm for W90A (blue) and wild-type (red) FL-PAC. The fitted curves using a single exponential function for W90A and a double-exponential function for FL-PAC are shown with black dashed lines.

**SI-7.** **Catalytic activity** **measurements**

To measure the light-dependent catalytic activity of FL-PAC, its absorption spectra were recorded at various light intensities in the absence of ATP and MaLionR (Figure 7a in the main text). The absorbance at 495 nm was used to determine the fraction of red-shifted species (*f*_red_) at each light intensity, using Eq. 1 in the main text (Figure S7a, left). Next, a mixture of Mg-ATP and MaLionR was added to the solution to initiate the enzymatic reaction and observe its progress (Figure S7a, right). The intensity of the illumination light was maintained to keep *f*_red_ constant during the measurement. ATP consumption was monitored as the time-dependent change in absorbance at 570 nm.

The absorption spectrum of MaLionR depended on ATP concentration (Figure S7b). To accurately determine the ATP concentration in the solution based on the absorbance of MaLionR, a calibration curve was generated by plotting the absorbance at 570 nm against known ATP concentrations (Figure S7c). The curve shows saturation at higher concentrations, but within the range of 0 – 300 μM ATP, it exhibited an almost linear relationship. This calibration curve was used to calculate the ATP concentration from the absorbance measurements.

The enzymatic activity was measured under various light intensities. The absorbance of MaLionR at 570 nm decreased as the reaction progressed, and these data were converted to ATP concentrations using the calibration curve (Figure 7b in the main text). The influence of the Michaelis constant (*K*_M_) can be disregarded in the region where the substrate concentration changes linearly with time. In this linear range, the reaction rate is directly proportional to the substrate concentration, indicating that the enzyme operated under conditions of substrate saturation. Therefore, the *K*_M_ value, which represents the substrate concentration at which the reaction rate is half of the maximum rate, becomes less significant in this range. Hence, the rate of ATP consumption was calculated from the slope of the plot within this time range, which is above 150 μM of ATP (Figure 7b in the main text). Finally, using a concentration of FL-PAC (15 μM), the catalytic rate constant (*k*_cat_) was calculated for each condition and is plotted against *f*_red_ in Figure 7c in the main text.


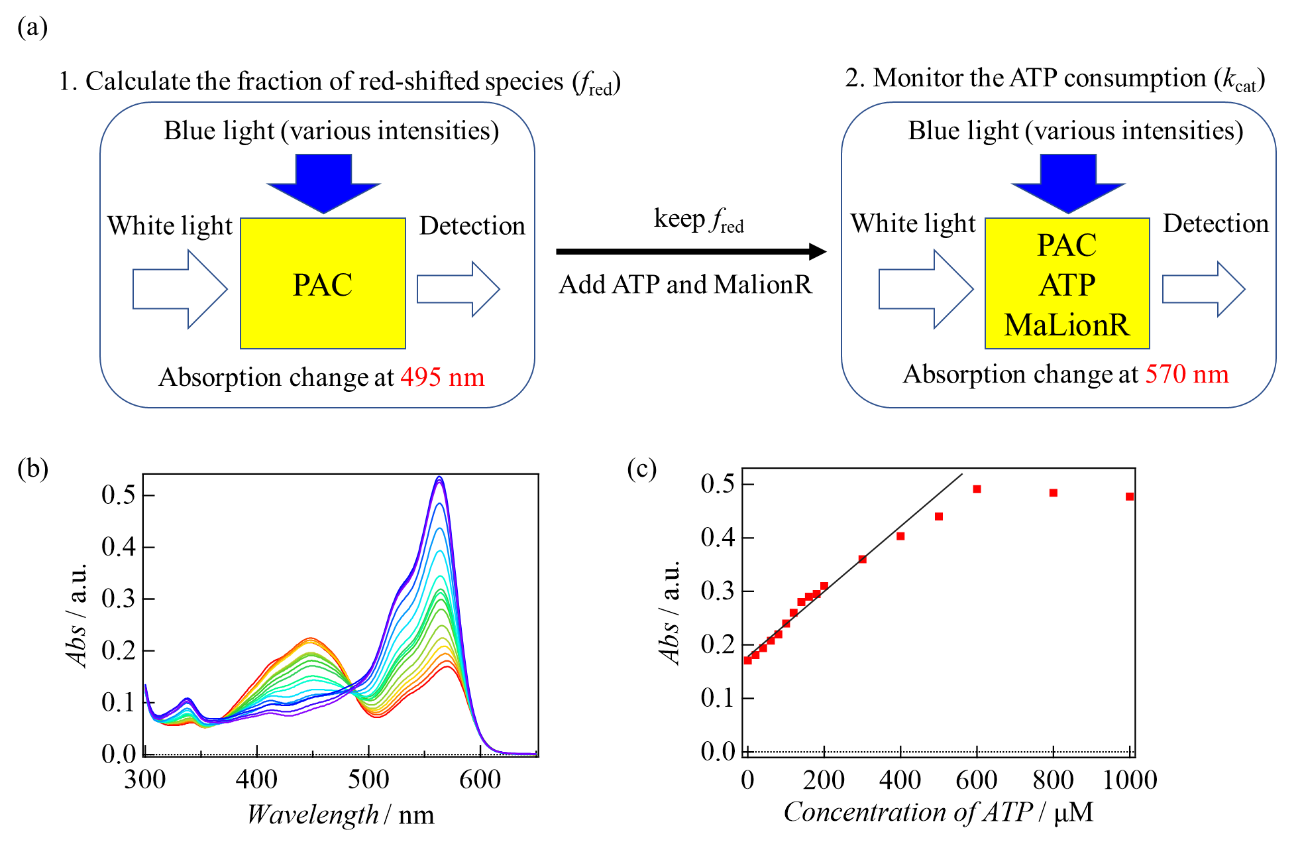


Figure S7. (a) Schematic illustration of the experimental procedure. (b) Dependence of ATP concentration on the absorption spectra of MaLionR. ATP concentrations ranged from 0 – 1,000 μM. Higher concentrations indicated by a transition from red to blue color in the data. (c) Relationship between the absorbance at 570 nm and the concentration of ATP. Within the range of 0 – 300 μM ATP, the data are fitted with a linear function, shown with a black solid line.

**SI-8.** **Amphiphatic character of α3-helix**


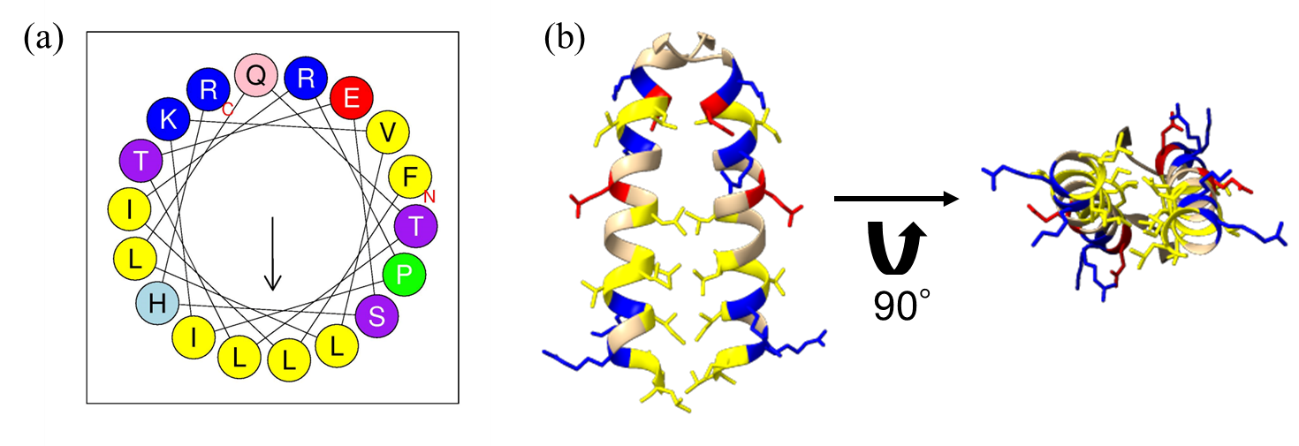


Figure S8. (a) Helical wheel analysis of the α3-helix of OaPAC. The C-terminal flanking 18 residues of the BLUF domain were analyzed using online software for helical wheel projection, available at: http://lbqp.unb.br/NetWheels/. Acidic residues, basic residues, and nonpolar residues are shown in red, blue, and yellow, respectively. (b) Mapping of acidic residues (red), basic residues (blue), and nonpolar residues (yellow) on the structure of the α3-helix extracted from the crystal structure of OaPAC in the dark (PDB ID: 4yut).

**SI-9.** **Aromatic residues located on the dimer interface**


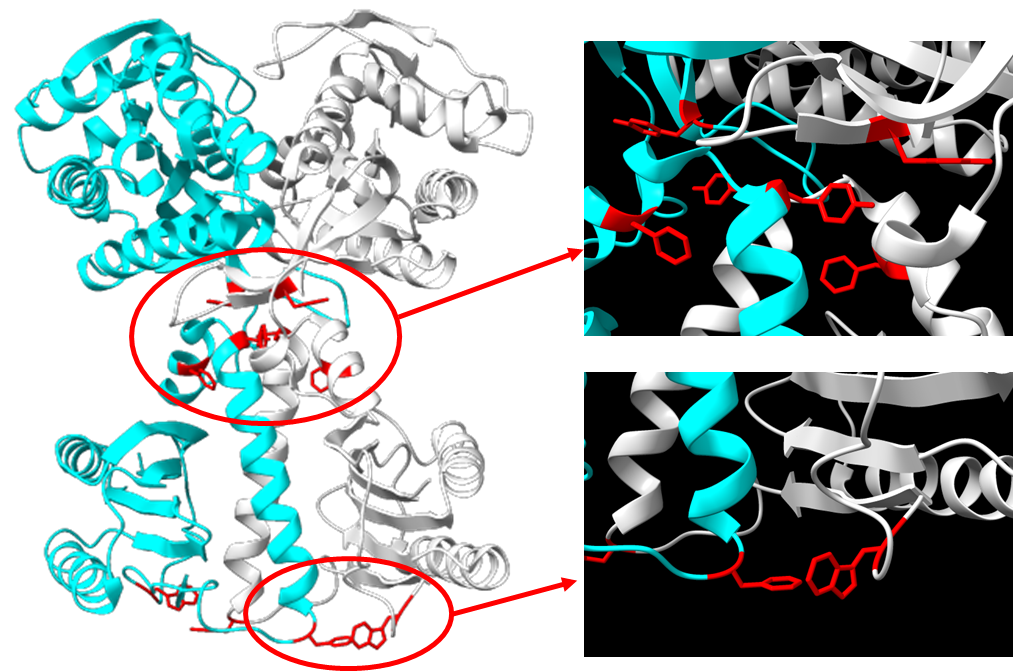


Figure S9. The two monomers in the dimer are shown in gray and cyan for clarity, and the aromatic residues near the dimer interface are displayed as red sticks. These residues may contribute to the light-induced CD changes in the near-ultraviolet region. The enlarged structures show aromatic residues sharing intersubunit hydrogen bonds (upper panel) and the interaction between the BLUF domain and the α3-helix (lower panel).
